# Advantages of imaging photoplethysmography for migraine modeling: new optical markers of trigemino‐vascular activation in rats

**DOI:** 10.1186/s10194-021-01226-6

**Published:** 2021-04-01

**Authors:** Alexey Y. Sokolov, Maxim A. Volynsky, Valery V. Zaytsev, Anastasiia V. Osipchuk, Alexei A. Kamshilin

**Affiliations:** 1 Department of Neuropharmacology, Valdman Institute of Pharmacology, Pavlov First Saint Petersburg State Medical University, Saint Petersburg, Russia; 2 Laboratory of Cortico-Visceral Physiology, Pavlov Institute of Physiology of the Russian Academy of Sciences, Saint Petersburg, Russia; 3 Faculty of Applied Optics, ITMO University, Saint Petersburg, Russia; 4 Research Laboratory of Neuromodulation, Almazov National Medical Research Centre, Saint Petersburg, Russia; 5 Laboratory of New Functional Materials for Photonics, Institute of Automation and Control, Russian Academy of Sciences, Vladivostok, Russia

**Keywords:** Migraine, Trigemino‐vascular system, Animal model, Intracranial blood flow, Imaging photoplethysmography, Electrical stimulation, Sumatriptan, Valproic acid

## Abstract

**Background:**

Existent animal models of migraine are not without drawbacks and limitations. The aim of our study was to evaluate imaging photoplethysmography (PPG) as a method of assessing intracranial blood flow in rats and its changes in response to electrical stimulation of dural trigeminal afferents.

**Methods:**

Experiments were carried out with 32 anesthetized adult male Wistar rats. Trigeminovascular system (TVS) was activated by means of electrical stimulation of dural afferents through a closed cranial window (CCW). Parameters of meningeal blood flow were monitored using a PPG imaging system under green illumination with synchronous recording of an electrocardiogram (ECG) and systemic arterial blood pressure (ABP). Two indicators related to blood-flow parameters were assessed: intrinsic optical signals (OIS) and the amplitude of pulsatile component (APC) of the PPG waveform. Moreover, we carried out pharmacological validation of these indicators by determining their sensitivity to anti-migraine drugs: valproic acid and sumatriptan. For statistical analysis the non-parametric tests with post-hoc Bonferroni correction was used.

**Results:**

Significant increase of both APC and OIS was observed due to CCW electrical stimulation. Compared to saline (*n* = 11), intravenous administration of both the sumatriptan (*n* = 11) and valproate (n = 10) by using a cumulative infusion regimen (three steps performed 30 min apart) lead to significant inhibitory effect on the APC response to the stimulation. In contrast, intravenous infusion of any substance or saline did not affect the OIS response to the stimulation. It was found that infusion of either sumatriptan or valproate did not affect the response of ABP or heart rate to the stimulation.

**Conclusions:**

Imaging PPG can be used in an animal migraine model as a method for contactless assessment of intracranial blood flow. We have identified two new markers of TVS activation, one of which (APC) was pharmacologically confirmed to be associated with migraine. Monitoring of changes in APC caused by CCW electrical stimulation (controlling efficiency of stimulation by OIS) can be considered as a new way to assess the peripheral mechanism of action of anti-migraine interventions.

## Background

Migraine is a form of primary headache associated with autonomic and sensory disturbances [1]. This disabling disease significantly reduces the quality of life, aggravates the course of comorbid pathologies and is a risk factor for the development of cardiovascular accidents [2–4]. Migraine is widespread in the world population and is a global social problem, causing significant damage to the economies of different countries due to direct and indirect material losses [3, 5–7]. Migraine treatment implies an alleviation and prevention of headache attack for which both medicinal and non-drug-induced approaches are used [8–10]. Unfortunately, the quality of this treatment leaves much to be desired and, despite the noticeable progress in the management of this cephalalgia and the expansion of the arsenal of specific drugs [11, 12], there remains a high unmet need for effective and safe methods of anti-migraine therapy [13, 14]. It is clear that the search for new treatments requires a preclinical phase. During this phase, experiments based on animal migraine models, which allow to transfer the experiment data to clinical practice with a certain degree of confidence, are rather productive [15–17].

The current understanding of migraine pathogenesis is associated with the hypothesis of the trigeminovascular system (TVS) activation, which is a key point taken together central neuronal and peripheral neuro-vascular pathophysiological events that determine the clinical picture of the disease [18, 19]. Consequently, the essence of majority of translational animal migraine models in vivo is to activate TVS by any exogenous trigger, either local (e.g., through electrical stimulation of dura mater [20]) or systemic (e.g., by NO donors injection [21]). Thereafter, TVS activation is to be assessed by any of the markers, among these could be increase of trigeminovascular neurons activity, decrease of cutaneous receptive fields mechanical threshold or increase of the diameter of meningeal arteries. Further monitoring and analysis of changes in the selected marker under the influence of any interventions studied (e.g., drug administration or exposure to physiotherapy) allows one to evaluate the therapeutic efficacy of the intervention [20, 22, 23]. Present-day animal migraine models are not without drawbacks and limitations, among which are low informativeness and efficiency, high cost of the equipment used, moderate predictability, and insufficient simulation power. All this necessitates the development of new methodical approaches in the experimental modeling of migraine [24–26], which will increase the output and quality of scientific products and will allow to intensify research in the field of cephalgology. The search and validation of new TVS activation markers is critical in these approaches [27–29], including using new technical ways of markers registration.

Imaging photoplethysmography (PPG) is a relatively simple, informative, and inexpensive method for assessing the parameters of tissue blood flow. This technique was demonstrated to be very effective in both identifying microcirculation features in migraine patients [30] and studying intracranial vascular reactions in laboratory animals in various experimental conditions [31, 32]. Therefore, the aim of our study was to evaluate imaging PPG as a method of assessing intracranial blood flow in rats and its changes in response to electrical stimulation of dural trigeminal afferents. Moreover, we intended to identify new, PPG-relative markers of TVS activation and to carry out pharmacological validation of these markers by determining their sensitivity to two clinically effective anti-migraine drugs: valproic acid and sumatriptan.

## Methods

### Animals and ethics

All experiments were performed in accordance with the ethical guidelines of the International Association for the Study of Pain, the Directive 2010/63/EU of the European Parliament and of the Council on the protection of animals used for scientific purposes, and reported in compliance with the ARRIVE guidelines 2.0. The study protocol was approved by the Institutional Animal Care and Use Committee of Pavlov First St. Petersburg State Medical University before carrying out the experiments. All efforts were made to minimize animal suffering and to use only the number of experimental subjects necessary to produce reliable data. Adult (mean body weight 452 ± 66 g, n = 32) male Wistar rats were purchased from the State Breeding Farm ‘‘Rappolovo” (Saint Petersburg, Russia). The animals were kept in groups (2–5 per cage) under standard laboratory conditions (12-h light/dark schedule) with food and water available ad libitum.

### Anesthesia and surgical preparation

Rats were anesthetized by intraperitoneal injection with a mixture of urethane (Sigma, St. Louis, MO, USA) and a-chloralose (Sigma, St. Louis, MO, USA) at an initial dose of 800/60 mg/kg. After achieving surgical anesthesia, each rat was placed on a thermostatically controlled heating pad, providing a constant body temperature. The trachea was intubated to measure a respiratory airflow and end-tidal carbon dioxide. The right femoral artery and vein were cannulated for continuous monitoring of arterial blood pressure (ABP) and drug administration, respectively. The animal’s head was fixed in a stereotax (Stoelting Co., Wood Dale, IL, USA) for further surgery aimed at forming a closed cranial window (CCW). It was achieved by thinning parietal bone by a micro-drill to the state of a thin membrane until the meningeal vessels were clearly visible through the remaining intact bone. During the surgery, all the tissues were cooled with the local use of cold saline solution. After the parietal bone drilling, the rat rested for at least 40 min to minimize the effect of postsurgical reaction.

**Fig. 1:**
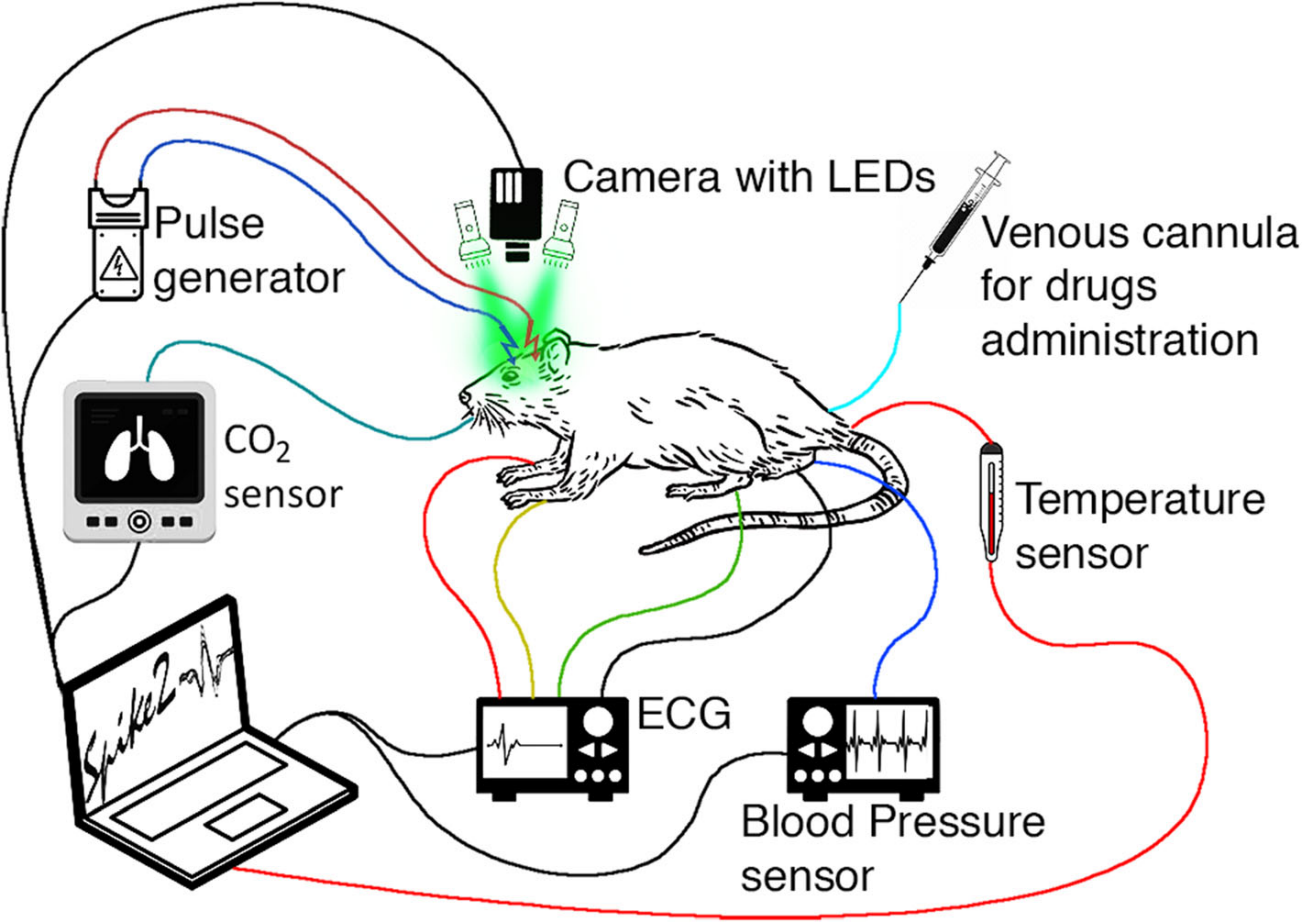
Schematic diagram of the experimental setup for monitoring the meningeal-blood-flow response and key physiological parameters to CCW electrical stimulation

Layout of the experimental setup for monitoring the response of both the key physiological parameters and meningeal blood flow to CCW electrical stimulation is shown in Fig. 1. Stimulation electrodes (two varnish insulated silver wires) were placed on the CCW surface nearby visible blood vessels. The CCW area was covered with mineral oil to reduce tissue dehydration and increase the transparency of the residual bone for continuous video recordings of intracranial vessels. Steel needles were inserted into the muscle tissue of the rat limbs to record electrocardiograms (ECGs) during the experiment. Digital electrocardiograph (model KAP-01-“Kardiotekhnika-EKG,” Incart Ltd., Saint Petersburg, Russia) operating at the sample frequency of 1 kHz was used to record ECG synchronized with video frames with one millisecond accuracy. Throughout the experiments, rectal temperature was monitored and maintained within a range of 37–38 °C. Continuous monitoring of ABP and end-tidal CO_2_ were carried out by the pressure sensor (MLT844, AD Instruments Inc., Colorado Springs, USA) and carbon dioxide analyzer (Capstar-100, CWE, Inc., Ardmore PA, USA), respectively. These data were digitized at the sample frequency of 10 kHz (ADC-DAC Power1401-3, Cambridge Electronic Design, Cambridge, UK) and recorded in the personal computer using Spike2 version 8 software (Cambridge Electronic Design, Cambridge, UK). The adequacy of anesthesia was judged by the stability of the arterial blood pressure and respiratory airflow in the absence of noxious stimulation. Supplementary anesthetic was administered through the venous catheter when necessary. In some cases, the animals were paralyzed with pipecuronium bromide (1,0 mg/kg initially; maintained with 0.4 mg/kg as required, i.v.; ‘‘Arduan”, Gedeon Richter, Budapest, Hungary) and artificially ventilated with room air (SAR-830, CWE, Inc., Ardmore PA, USA). After the end of each experiment, the rat was euthanized by intravenous injection of a lethal dose of urethane (3 g/kg).

### Optical imaging and experimental protocol

A custom-made imaging PPG system used in these studies for monitoring meningeal blood flow was similar to what has previously been reported [32]. An optical unit indicated in Fig. 1 as “Camera with LEDs” consisted of a digital camera with a standard lens of 25-mm focal lens and illumination block. Light at the wavelength of 530 ± 25 nm was provided by eight light-emitting diodes (LEDs) with the flux of 100 lm per diode. All the LEDs were mounted around the camera lens. The camera lens and LEDs were covered by cross-oriented film polarizer (Edmund Optics, 0.18 mm thickness) to increase the signal-to-noise ratio (SNR) of blood-vessels imaging [33]. A digital monochrome camera with complementary metal-oxide-semiconductor sensing matrix (10-bit model GigE uEye UI-5220SE of the Imaging Development System GmbH) acquired CCW images. The optical unit was placed 15 cm above the rat’s head (see Fig. 1) providing focused CCW images of 16.3 × 10.4 mm^2^. Videos were recorded at 100 frames per second with a resolution of 752 × 480 pixels and were saved frame-by-frame on the hard disk of a personal computer.

All studied animals (n = 32) were divided into 3 groups. In the first group of rats (n = 10), we tested the effect of cumulative intravenous infusion (three steps performed 30 min apart) of valproate (Depakine, Sanofi, France; 100 mg/kg per step) on the response of meningeal blood flow to CCW stimulation. The second group (n = 11) received cumulative intravenous infusion (in three similar steps) of sumatriptan (Tokyo Chemical Industry Co., Ltd., Japan; 4 mg/kg per step), whereas the third group (n = 11) received an injection of isotonic saline in the same regime and volume (0.7 ml per step), as the other two, and served as a control.

In the baseline stage (before any injection), we recorded changes in meningeal blood flow in response to CCW electrical stimulation, while monitoring changes in physiological parameters (ABP, heart rate, HR, and end-tidal CO_2_). To this end, video of visible intracranial tissues was recorded by the imaging PPG system continuously in the pre-stimulation phase (20 s), during stimulation (15 s), and afterwards (85 s), with the total duration of 120 s for each trial. Bipolar electrical stimulation was delivered by a computer-controlled stimulator (Master-8, A.M.P.I., Jerusalem, Israel) connected to a stimulus isolation unit. Biphasic trains of 2 ms rectangular pulses were delivered at 10 Hz for 15 s at an amplitude of 50 V by means of the stimulation electrodes in contact with CCW (see Fig. 1). Hemodynamic response to the CCW stimulation in the baseline was measured three times every 10 min for each rat (triple trials). Thereafter, by analogy with measurements in the baseline stage, the reaction of vasculature to the stimulation was also assessed three times every 10 min (triple trials) after each infusion of the testing substance or saline. The total duration of the experiment with one rat was more than 120 min.

### Data processing

All recorded video frames, ECG and ABP data were processed off-line by using custom software implemented in the Matlab platform (Version R2018b, The MathWorks, Inc., MA, USA, 2018). Pixel values of the recorded frames were measured to estimate two indicators related to parameters of tissue blood flow, namely optical intrinsic signals (OIS) and amplitude of the pulsatile component (APC) of the PPG waveform. As well known, light reflected from a living tissue is modulated in time due to dynamic changes in the optical properties of the tissue itself [34, 35]. These changes consist of a pulsating component synchronized with the heart rate and a slowly varying component (in the time scale of few seconds) associated with changes in tissue blood volume. The latter component is used to assess OIS. OIS imaging technique enables visualization of functional changes in cerebral blood volume, particularly, due to electrical stimulation [36, 37]. Temporal modulation of OIS at the heartbeat frequency is typically considered as the noise that have to be filtered out to increase SNR [38, 39]. In contrast, heartbeat related modulation is the primary source of information in imaging PPG systems [40]. Visualization of hemodynamic changes in rat’s cortex caused by cortical spreading depression by simultaneous use of OIS and PPG imaging revealed significant difference in spatial-temporal variations between OIS and APC parameters of the blood flow [41]. Although the hardware of imaging PPG is the same as for OIS imaging, the data processing in these systems is different.

Motion artifacts are the most significant sources of noise when extracting hemodynamic parameters from recorded videos. Sometimes, to achieve a high SNR in OIS imaging, a glass plate was placed on the surface of the brain to attenuate tissue movement [36, 39]. In our calculations, we applied digital image stabilization algorithm to compensate tissue motion described in our recent paper [42]. Considering stochastic and heterogeneous motion of different parts of the cortex, the entire image frame was divided into 16 × 16 pixel segments, with subsequent compensation for the motion of each segment independently. We assume that the modulation of the reflected light intensity (leading to changes in pixel values) contains two components, one of which is caused by a change in the blood volume interacting with light, and the other is due to tissue motion. The motion-related component is proportional to the image gradient and lateral offset. The offset was estimated in every segment by optical flow algorithm using gradient method [43]. Thereafter, the motion-related component was reconstructed and subtracted from the original signal. Both OIS and APC were estimated from the motion-compensated frames.

**Fig. 2:**
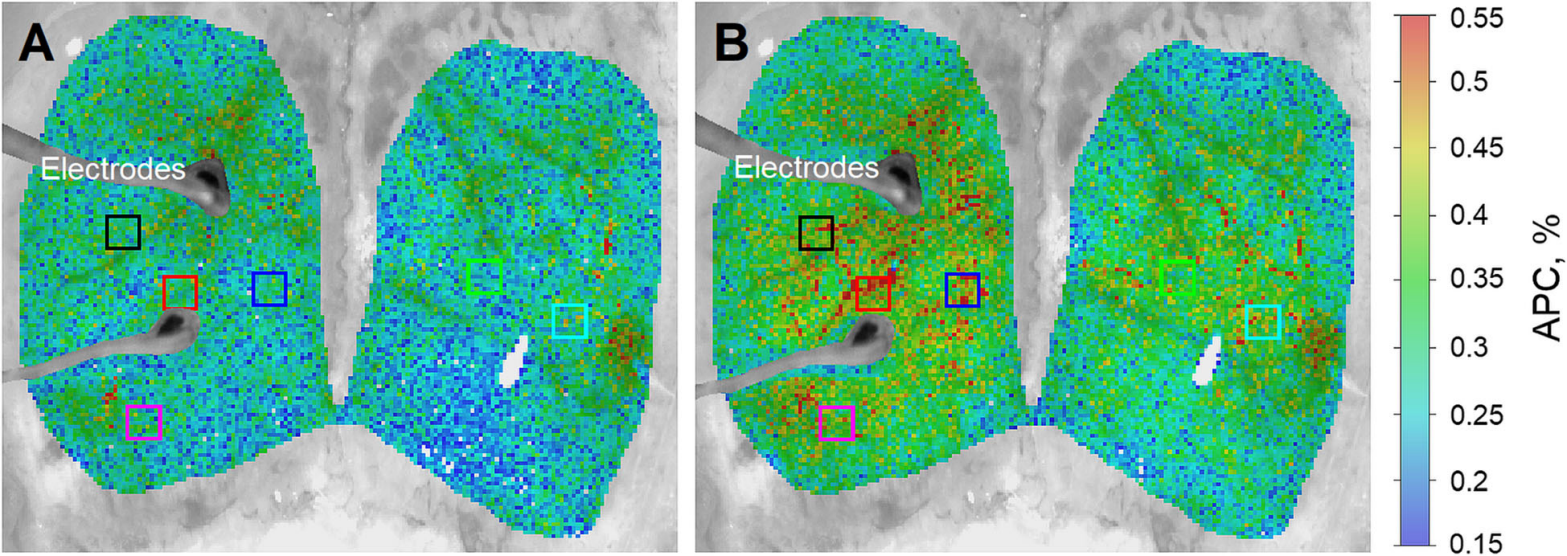
Pseudo-colored maps of the electrical stimulation-evoked changes in APC over the cortical surface at 530 nm. **a** Spatial distribution of APC overlaid with the initial cortex image as estimated at the beginning of the pre-stimulation phase (10th s of the trial). **b** APC mapping during stimulation (30th s of the trial). The color bar on the right shows APC as a percentage, which is the same for both panels (**a**) and (**b**). Colored squares show the position of selected regions (big regions of interest) in which changes in mean APC were assessed to monitor the parameter’s dynamics

To assess APC, we calculated PPG waveforms as frame-by-frame evolution of the mean pixel value in every small region of interest (ROI) sizing 3 × 3 pixels that corresponds to an area of 65 × 65 µm^2^ at CCW. Time boundaries for each heartbeat were determined using R-peaks of a synchronously recorded ECG. The waveform was calculated as the ratio of the alternating component (AC) and slowly varying (DC) component (AC/DC ratio). The latter one was used for estimation of OIS. Then, a mean PPG pulse was calculated by averaging the waveforms of 10 subsequent cardiac cycles defined by R–R intervals. The parameter APC was calculated as difference between the maximum and minimum values of the mean PPG pulse. Since APC was estimated in every small ROI, it allows us to mapping this parameter over the rat’s cortex. Representative examples of calculated APC maps, which illustrate an effect of CCW electrical stimulation for one of the rats, are shown in Fig. 2. It is clearly seen that the APC parameter increases throughout the entire cortex, while large pulsating arteries are clearly distinguished from large veins because the arteries have much higher amplitude of the pulsatile component synchronized with cardiac activity.

OIS was calculated as difference in images between the current moment and at the beginning of each trial using the following equation:

1 $${OIS}_{i}\left(x,y\right)=\frac{{I}_{0}\left(x,y\right)-{I}_{i}\left(x,y\right)}{{I}_{0}\left(x,y\right)}100\%.$$

Here, ${I}_{0}\left(x,y\right)$ is the first image at the pre-stimulation phase in each trial, and ${I}_{i}\left(x,y\right)$ refers to the image at the i’th time-point. The parameter determined in this way increases with increasing cerebral blood volume [36, 37]. As known, electrical stimulation of brain evokes significant local changes in OIS in the vicinity of the electrodes under illumination at 535 or 546 nm reflecting neurogenic vasodilation [36, 39]. Since the calculation of the OIS parameter is quite fast, we used it to control the electrical contact with CCW: if the increase in OIS during stimulation was less than 7 %, then the contact was mechanically adjusted. OIS parameter was also used to quantify changes in meningeal blood volume caused by electrical stimulation in each trial.

For a quantitative assessment of the dynamics of changes in meningeal blood flow, six big ROIs with a size of 27 × 27 pixels corresponding to 0.6 × 0.6 mm^2^ at the rat’s cortex were selected. Their position was chosen on the APC map calculated at the 10th sec of the stimulation, in places with the highest amplitude of the pulsatile component. An example of the location of these big ROIs for one of the rats is shown in Fig. 2. Two of them (e.g., the red and black in Fig. 2) were selected in vicinity of the electrodes and contained large arteries. In all trials, the greatest stimulation-induced change in APC was always observed near the electrodes. Consequently, these two ROIs were used to monitor changes in meningeal blood flow as the most representative. In each big ROI, an average was calculated for both APC and OIS.

To compare the hemodynamic parameters (APC, OIS, ABP, and HR) of different animals, we estimated the degree of their electrostimulation-induced change relative to the pre-stimulation phase. To this end, each parameter was normalized to its mean during the pre-stimulation phase (the first 20 s of each trial). Normalized parameters are expressed as a percentage, assuming a pre-stimulation level of 100 %. The normalization was carried out individually for each experimental trial both before (during the baseline) and after every administration of testing substance (either sumatriptan or valproate) or saline. To assess changes in APC and OIS responses to electrical stimulation due to pharmacological interventions, we adopted parameter that is an integral difference (in respect to the pre-stimulation phase) in APC or OIS calculated during the stimulation phase (between 20 and 35 s for every trial).

### Statistical analysis

A total of 384 trials were carried out with 32 rats: there were 3 trials in the baseline stage for each rat, and 3 trials repeated 3 times after every infusion of either the testing substance or saline. Considering the results of the Kolmogorov-Smirnov test of the data distribution normality, we estimate how each infusion of a testing substance or saline affects the assessed hemodynamic parameters by means of either the non-parametric Wilcoxon signed-rank test and Friedman test (for paired samples) or Kruskal-Wallis test (for unpaired samples) with post-hoc Bonferroni correction. Statistical significance was set at *p* < 0.05. The experimental data are presented in the following figures as median values with interquartile ranges unless otherwise indicated in the text. Analysis and graphical presentation of the results was carried out using the Matlab software (Version R2018b, The MathWorks, Inc., MA, USA, 2018).

## Results

### Initial characteristics of systemic hemodynamic

As measured in the first pre-stimulation phase during the baseline stage, the studied animals (*n* = 32) had the following physiological parameters. The mean ABP was 72 ± 17 mmHg (Mean ± SD) while the heart rate was 413 ± 44 beats per minute. The indices of systemic hemodynamics in animals of the two main and control groups did not differ significantly (for ABP, *p* = 0.494, KW = 1.41; for HR, *p* = 0.288, KW = 2.49, Kruskal-Wallis test).

### Effect of electrical stimulation of the closed cranial window on PPG parameters

**Fig. 3:**
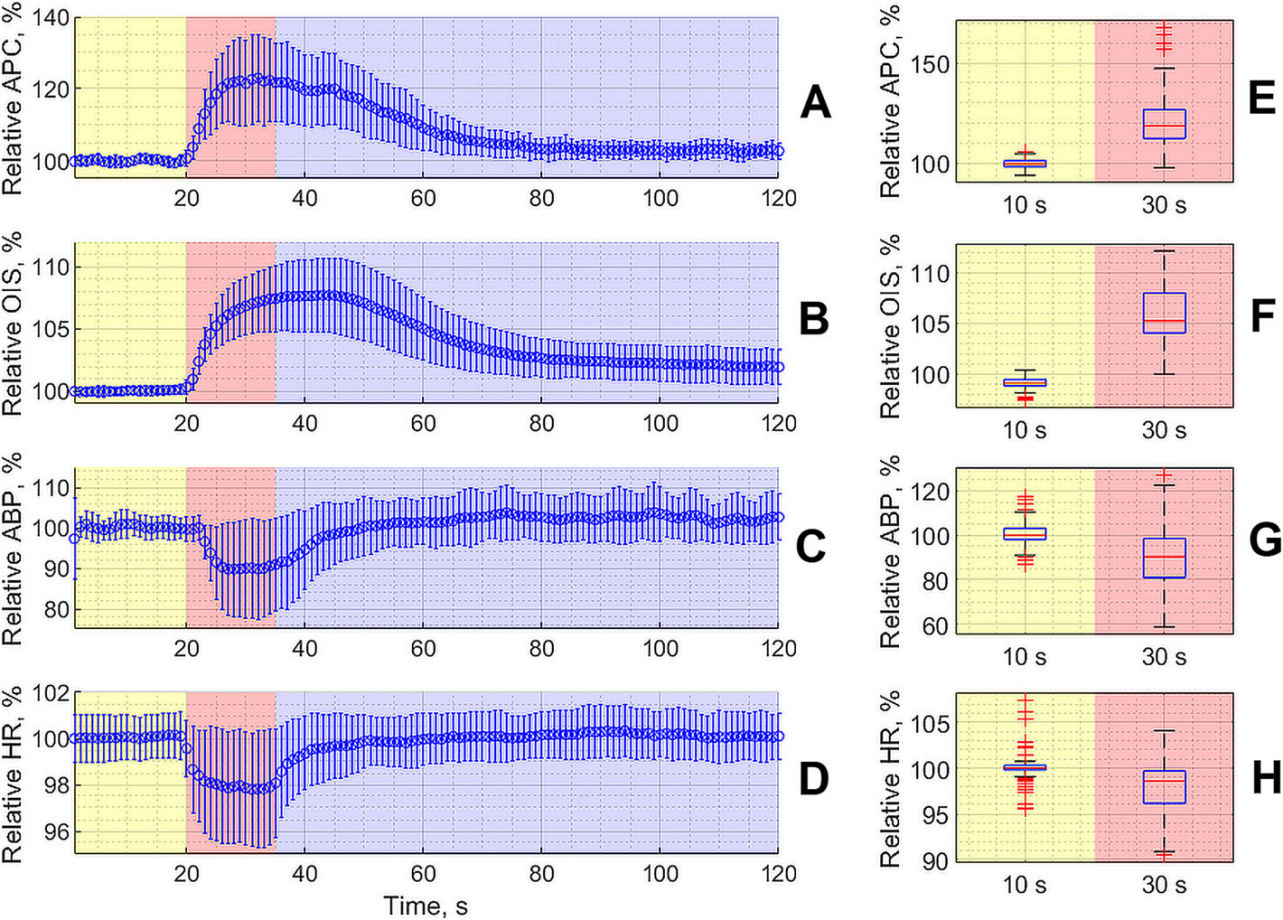
Relative change of the parameters measured in a contactless way by the digital camera (APC, graph **a**, and OIS, graph **b**) and invasively (ABP, graph **c**, and HR, graph **d**) in response to the CCW electrical stimulation. Here we show the data averaged over 96 baseline measurements carried out in three subsequent trials with three groups of animals before pharmacological interventions. Curves in the graphs show evolution of the mean value while the whiskers are the standard deviations. Yellow color indicates the pre-stimulation phase, the red one marks a 15-seconds period of the electrical stimulation, and the violet is the relaxation phase. Right-side panels (**e**, **f**, **g** and **h**) illustrate the statistical significance of the respective hemodynamic parameter assessed at the 10th s of the trial (pre-stimulation phase) and at the 30th s of the trial (stimulation phase)

Response of both optically (APC and OIS) and invasively (ABP and HR) measured parameters of the meningeal blood flow and systemic hemodynamics, respectively, to CCW electrical stimulation during the baseline stage (before any pharmacological intervention) is shown in Fig. 3. Each electrical stimulation of the dural afferents through CCW resulted in significant increase of both the APC (Fig. 3 a) and OIS parameters (Fig. 3b) for all 32 rats (*p* < 0.001 for both parameters, Wilcoxon signed-rank test, Fig. 3e f). As seen, an increase of optically assessed parameters occurs immediately with the beginning of the stimulation and continues after its end with slow relaxation. Such an increase was accompanied by transient diminishing of both ABP and HR as shown in Fig. 3 c, 3g and 3d, 3 h, respectively. Nevertheless, these parameters returned to their respective pre-stimulation values much faster than APC or OIS. It is worth noting that the time to reach the maximum APC increase was shorter than for OIS.

**Fig. 4:**
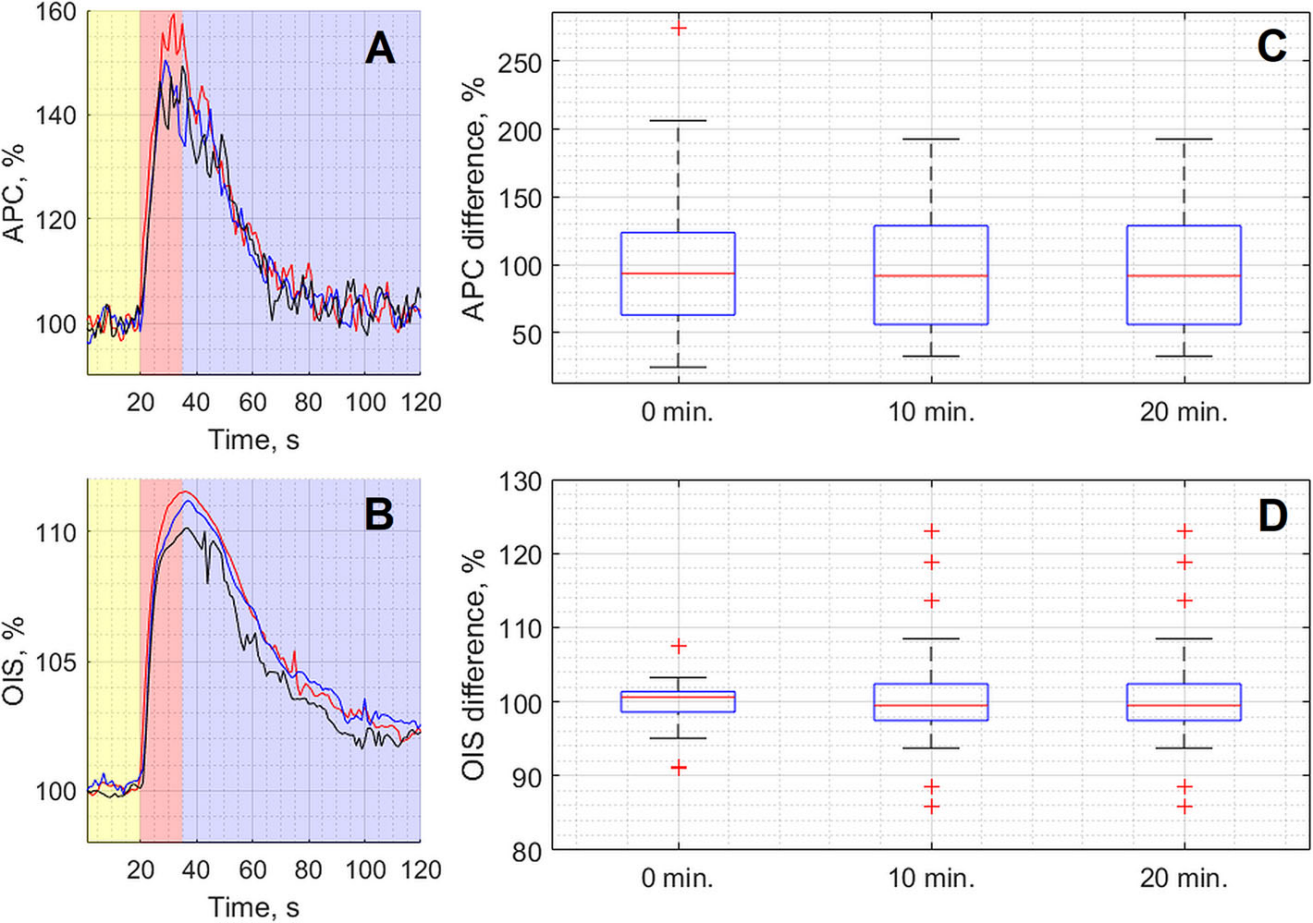
Representative example of the responses of optical parameters APC (panel **a**) and OIS (panel **b**) to three subsequent CCW electrical stimulations repeated every 10 min for one of the rats during the baseline stage. Each curve in panels **a** and **b** shows dynamics of APC and OIS parameters averaged for the selected two most representative ROIs. The red curve was obtained in the first trial (0 min), the blue in the second trial (10 min), and the black in the third trial (20 min). The box plots in the panels **c** and **d** show cumulative changes in the responses to the stimulation of APC and OIS, respectively. In the panels **c** and **d**, the response was estimated as an integral of the respective curve shown in graph **a** or **b** (see details in the text). The data were obtained every 10 min for all rats (*n* = 32) in the baseline stage (before pharmacological interventions)

Typical example of the APC and OIS dynamics recorded in the baseline stage for one of the rats is shown in Fig. 4. One can see that changes in these parameters were reproducible over time, i.e. they consistently occurred during repeated stimulation with a 10-minute interval between stimulations. Quantitatively, the response of the analyzed parameters to electrical stimulation was estimated as the integral under the curve (see, for example, Fig. 4 a-b). The integration period was chosen for each animal individually, starting from the 20th s of each trial (the beginning of the stimulation phase) and ending at the moment when the parameter was returned to its value ​​during the pre-stimulation phase with an accuracy of 2 SD. On average, the integration period was 65 s varying from 40 s to 90 s. Further, for the generality of data presentation for each animal, the calculated integrals were normalized to the average value for all three trials in the baseline. It was found that the response of APC or OIS to the stimulation had statistically insignificant differences among triple stimulations: for APC, *p* = 0.804, *F* = 0.44; for OIS, *p* = 0.216, *F* = 3.06, Friedman test.

### Effect of pharmacological interventions on the APC response to CCW electrical stimulation

**Fig. 5:**
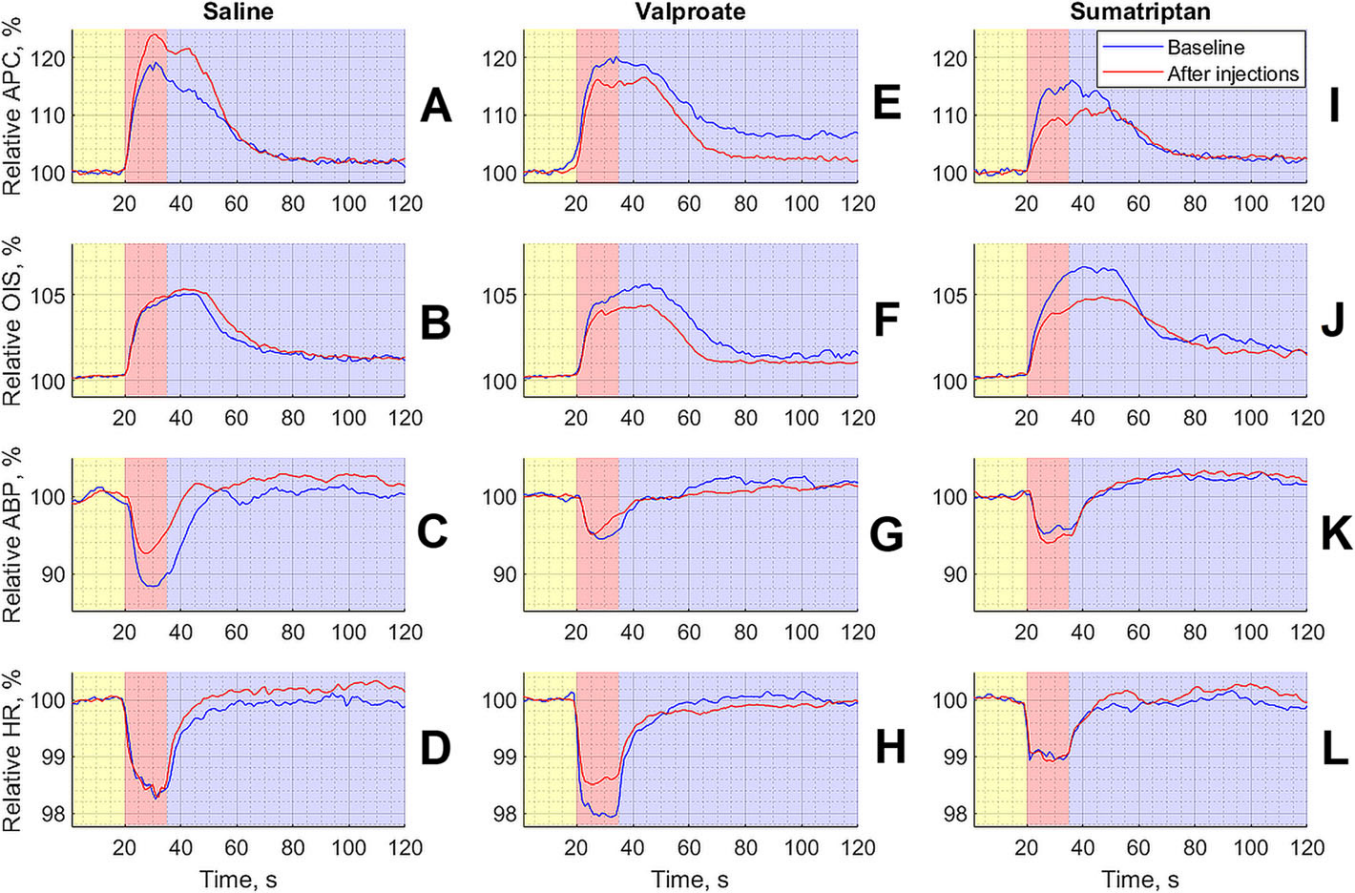
Summarized responses of both PPG and hemodynamic parameters to CCW electrical stimulation averaged over studied animals separately in each of three groups. Panels **a** – **d** show responses for the group that received infusion of saline, panels **e** – **h** for the group of valproate, and panels **i** – **l** for the group of sumatriptan. In all panels, yellow, red, and violet highlighting indicates pre-, during-, and post-stimulation phase, respectively. The time scale in seconds is shown in X-axis of each graphs, whereas the normalized parameters in a percentage are shown in Y-axes. The blue curve in each graph is the response of the measured parameter during the baseline stage, and the red curve is the response after an infusion of a respective substance. All curves were calculated as mean over three steps of the substance infusions

**Fig. 6:**
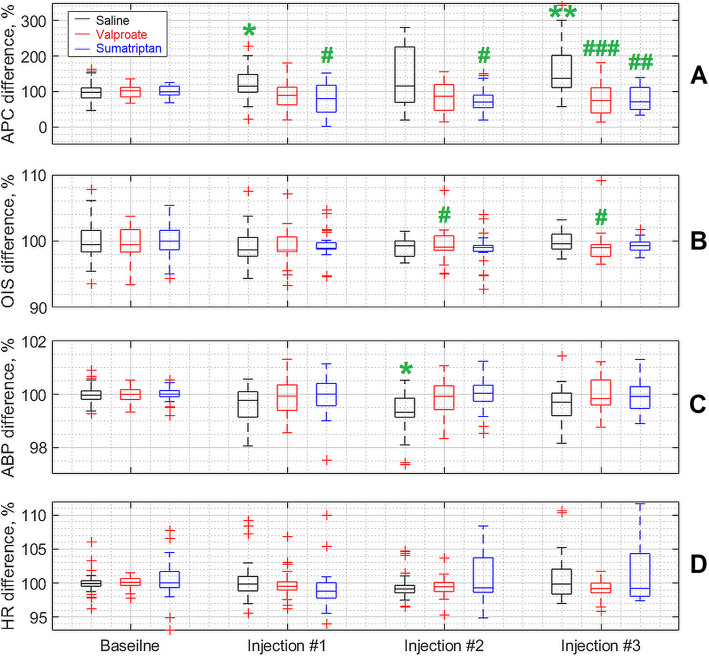
Dynamics of changes in parameters of systemic hemodynamics and PPG due to CCW electrical stimulation. Each box plot is a composite of indicators of a specific parameter for a 30-minute interval before (baseline stage) and after each of three infusion steps of the testing substance. The black, red and blue boxes correspond to the saline, valproate and sumatriptan infusion group, respectively. Green stars indicate statistical differences in relation to the baseline stage, whereas green grids represent differences between testing substance and saline. One symbol corresponds to *p* < 0.05, two of them correspond to *p* < 0.01, and three of them correspond to *p* < 0.001. All statistical significance is indicated with Bonferroni corrections for multiplicity

Intravenous administration of saline, sumatriptan, or valproate had different effects on the response of the measured parameters to electrical stimulation. The summary curves of the changes in both optically (APC and OIS) and invasively (ABP and HR) measured hemodynamic parameters in three groups of animals that received different pharmacological interventions are shown in Fig. 5. Intravenous infusion of saline in the cumulative regime (three subsequent injections with the interval of 30 min) resulted in significant increase of APC response to CCW electrical stimulation (*р* = 0.002, *F* = 15.07, Friedman test) as shown in Fig. 5a. This reaction was steadily increasing with the time so that the post-hoc Bonferroni analysis has revealed significant difference between the baseline and after the first infusion step (*p* = 0.034), as well as between the baseline and after the third (*p* = 0.02) infusion step, see Fig. 6. In the same time, intravenous administration of both the sumatriptan and valproate by using the same infusion scheme lead to the slow inhibiting effect on the APC response to the CCW electrical stimulation. However, this effect did not reach the significance level: for both drugs it was *р* = 0.145, *F* = 5.4, Friedman test.

No differences among the groups were observed during the baseline stage (*p* = 0.999, KW = 0). Nevertheless, significant differences were revealed already after the first infusion (*p* = 0.020, KW = 7.8) that were enhanced after the second (*p* = 0.015, KW = 8.35) and the third (*p* = 0.0002, KW = 17.21) administration of both the drugs and saline according to the Kruskal-Wallis test. The post-hoc Bonferroni analysis has revealed statistically significant difference between the groups of saline and sumatriptan (*p* = 0.020) after the first infusion, again between the same groups after the second infusion (*p* = 0.019). Whereas, the third infusion revealed difference between the saline and valproate groups (*p* = 0.0007), and between the saline and sumatriptan groups (*p* = 0.0015) as illustrated in Fig. 6.

### Effect of saline, sumatriptan, and valproate on the OIS dynamics

In contrast to APC, intravenous infusion of saline in a cumulative mode did not affect changes in OIS caused by electrical stimulation of CCW (*p* = 0.375, *F* = 3.11, Friedman test). Nevertheless, intravenous administration of sumatriptan by using the same scheme lead to a significant decrease in OIS response to electrical stimulation (*p* = 0.011, *F* = 11.07, Friedman test). However, post-hoc Bonferroni analysis did not reveal any significant differences in pairwise comparison (everywhere *p* > 0.05). Infusion of valproate also had a weak inhibitory effect on the OIS response, which, however, did not reach the level of significance (*p* = 0.465, *F* = 2.56, Friedman test), see Figs. 5 and 6.

Before drug or saline administration (during the baseline stage), no significant differences in the response of OIS to the electrical stimulation were revealed among the groups (*p* = 0.206, KW = 3.16) as it is shown in Fig. 6 b. Similarly, there were no differences after the first infusion (*p* = 0.054, KW = 5.84). However, the second and third infusions of either substance or saline lead to significant differences in OIS response between the groups: *p* = 0.033, KW = 6.8 and *p* = 0.028, KW = 7.12, respectively (everywhere the Kruskal-Wallis test). Post-hoc Bonferroni analysis revealed differences after the second and third infusions between the saline and valproate groups: *p* = 0.045 and *p* = 0.049, respectively (Fig. 6 b).

### Changes in parameters of systemic hemodynamics due to saline or drugs administration

Both the sumatriptan and valproate did not affect the response of ABP or HR to the CCW electrical stimulation as compared to the baseline stage (*p* > 0.05 for each infusion, Friedman test). In contrast, infusion of saline resulted in significant changes in the response both for ABP (*p* = 0.009, *F* = 11.65) and HR (*p* = 0.019, *F* = 9.91, Friedman test). In addition, post-hoc Bonferroni analysis revealed significant differences between the parameters after the second infusion and baseline for ABP (*p* = 0.014), and between the second and first infusions for HR (*p* = 0.014). Intergroup analysis did not reveal significant differences at all points in the time course for either BP or HR (all *p* > 0.05, Kruskal-Wallis test), see Fig. 6 c and 6 d.

## Discussion

In this experimental study, we have used the electrical stimulation of the dura mater through the closed cranial window that is well-studied and valid method to activate TVS [15]. On the one hand, it is well known that electrical stimulation of dura mater in humans is accompanied by an appearance of migraine-like headache [44–46]. On the other hand, it was shown time and again that such a stimulation causes an increase of metabolic and/or spike activity of 1st to 3rd order trigemino-vascular neurons, as well as neurogenic dilatation of dura mater arteries [20, 23, 47]. According to the trigemino-vascular theory, the abovementioned changes directly relate to the migraine pathogenesis [18, 19], and are to be considered in the experimental cephalgology as trustworthy markers of TVS activation [15]. In our experimental study, we have shown for the first time that electrical stimulation of dural trigeminal afferents through CCW resulted in significant change in PPG parameters assessed on visible intracranial tissues, namely, by an increase in APC and OIS signals. These changes were stable, reproducible, and accompanied by transient diminishing in both ABP and HR. Interestingly that ABP and HR restorations to the respective pre-stimulation level were much faster than for APC and OIS. These observations allows us to consider relative increases in APC and OIS as markers of TVS activation. However, relations of these markers to migraine are to be checked by validation of their sensitivity using clinically approved anti-migraine interventions. Valproic acid and sumatriptan for a long time are successfully used for preventive and abortive migraine treatment, respectively. Moreover, their efficacy many times was confirmed in clinical trials [8, 9]. It is well known that the previously indicated markers of TVS activation are sensitive to these drugs. In the experiment it was shown that valproate and sumatriptan, with different routes of administration, suppress the activity of TVS neurons and/or inhibit neurogenic dural vasodilatation [48–51]. Therefore, we have chosen valproate and sumatriptan as control drugs for testing a new potential marker of TVS activation.

We have demonstrated for the first time that saline and any substance (valproate or sumatriptan), when administered in a cumulative mode, did not lead to a change in the degree of OIS enhancement caused by CCW electrical stimulation. However, for valproate and sumatriptan a tendency was traced and consisted in some inhibition of the OIS changes caused by electrical stimulation. Ultimately, this trend led to the emergence of statistically significant differences between the groups of saline and valproate. However, hardly the revealed changes with a borderline significance level (p = 0.045 / 0.049) are essential from a physiological point of view.

Despite the fact that the OIS marker did not demonstrate any notable sensitivity to the test drugs, it is of undoubted interest as an indicator of the quality of electrical stimulation of trigeminal afferents, which makes it possible to exclude the receipt of fake results. It is known that OIS is proportional to the total blood volume in tissues [37], while the electrical stimulation leads to a local increase in the volumetric blood flow velocity, i.e., hyperemia in the irritated area [52–54]. By monitoring OIS during each trial of CCW electrical stimulation, it is possible to control the quality of the electrode contact with CCW, which is critical for efficient stimulation of trigeminal afferents. Absence of significant changes in OIS during stimulation indicates malfunction of the contact and suggests electrodes readjustment. Such a control practically excludes achievement of the false-positive results when anti-cephalalgic interventions are testing, thus improving quality of the scientific product.

Nonetheless, it was the saline, not pharmacological drugs, which significantly increases the degree of APC response to electrical stimulation of CCW. Neither valproate nor sumatriptan had significant influence on this parameter, whereas a tendency to suppressing of the APC increase was observed in the time course for the both groups. This fact suggests that the reaction of APC to stimulation is spontaneously increasing in the time course but such an increase is at least restrained or at maximum inversed by anti-migraine drugs. Indeed, the divergent dynamics of changes in the APC response to electrical stimulation caused by saline and drugs reaches the level of statistically significant differences after the first infusion and increases dramatically towards the end of the experiment. In that respect, sumatriptan has a noticeably more reliable and stably progressive effect than valproate.

Therefore, it can be stated that the APC marker (in contrast to OIS) has a certain sensitivity to anti-migraine drugs and can be used as an evaluation criterion of effectiveness in preclinical screening of potential anti-migraine interventions. It is worth noting that this sensitivity is low thus requiring selection of an adequate sample size. Moreover, a limitation of the APC marker is its ability to reflect only peripheral neurovascular events, but not central neuronal processes at the spinal and suprasegmental levels of the central nervous system that occur during TVS activation.

A possible explanation for the mechanism of the suppressive or, more accurately, the staying effect of drugs on the APC reaction is the increase in the rigidity of the vascular wall of the intracranial arteries. Walls rigidity increase (or increase of the vascular tone) results in diminishing of the blood pulsation amplitude [32], whereas the total blood volume in tissues does not change because OIS remains practically unchanged or even decreases slightly.

In general, this explanation is in the trend of conceptions about the peripheral neurovascular mechanism of the anti-migraine effect of sumatriptan: the drug is regarded as a cerebral vasoconstrictor [55] more precisely, it prevents excessive dilation of cranial arteries in a 5HT-1B/1D-agonistic manner [56]. As for valproic acid, the revealed “vascular” effect of the drug can be regarded as a new component of its anti-cephalalgic pharmacodynamics, although it manifests itself in the maximum dose. However, back in the mid-90 s of the last century, it was shown that valproate is able to inhibit vaso- and neurogenic plasma proteins extravasation in the dura mater through the mediation of GABA-A receptors [57], thereby reducing the severity of aseptic meningovasculitis, which is often presented as one of the components of migraine pathogenesis [18]. Nevertheless, a detailed clarification of the receptor mechanism of the inhibitory effect of valproate on the APC marker requires further research. It should be noted that the inhibitory effect of both drugs on the APC reaction was not associated with any of their influence on the dynamics of ABP and HR, which rather supports the local (i.e. at the level of intracranial vessels) implementation of this effect without involving systemic hemodynamics.

In conclusion, we have shown in our study that imaging PPG can be used in an animal migraine model as a method for assessing intracranial blood flow in rats and its changes in response to electrical stimulation of dural trigeminal afferents. We have identified two new markers of TVS activation, one of which (APC) was pharmacologically validated as migraine related, whereas the second (OIS) showed itself as an important element in controlling the quality of TVS activation in the experiment. Monitoring of changes in APC due to CCW electrical stimulation (under the control of OIS) can be considered as a new way to assess the peripheral mechanism of action of anti-migraine interventions.

## Data Availability

The datasets supporting the conclusions of this article are included within the article.

## Abbreviations

PPG: Photoplethysmography
TVS: Trigeminovascular system
CCW: Closed cranial window
ECG: Electrocardiogram
ABP: Arterial blood pressure
OIS: Optical intrinsic signals
APC: Amplitude of the pulsatile component
LED: Light-emitting diodes
SNR: Signal-to-noise ratio
ROI: Region of interest
AC: Alternating component
DC: Slowly varying component
HR: Heart rate
GABA: Gamma-aminobutyric acid
